# Genetic Analysis of Pediatric Primary Adrenal Insufficiency of Unknown Etiology: 25 Years’ Experience in the UK

**DOI:** 10.1210/jendso/bvab086

**Published:** 2021-05-11

**Authors:** Federica Buonocore, Avinaash Maharaj, Younus Qamar, Katrin Koehler, Jenifer P Suntharalingham, Li F Chan, Bruno Ferraz-de-Souza, Claire R Hughes, Lin Lin, Rathi Prasad, Jeremy Allgrove, Edward T Andrews, Charles R Buchanan, Tim D Cheetham, Elizabeth C Crowne, Justin H Davies, John W Gregory, Peter C Hindmarsh, Tony Hulse, Nils P Krone, Pratik Shah, M Guftar Shaikh, Catherine Roberts, Peter E Clayton, Mehul T Dattani, N Simon Thomas, Angela Huebner, Adrian J Clark, Louise A Metherell, John C Achermann

**Affiliations:** 1 Genetics and Genomic Medicine Research and Teaching Department, UCL Great Ormond Street Institute of Child Health, University College London, London, UK; 2 Centre for Endocrinology, William Harvey Research Institute, Queen Mary University of London, London, UK; 3 Children’s Hospital, Universitätsklinikum Dresden, Technische Universität Dresden, Dresden, Germany; 4 The Royal London Childrens Hospital, Barts Health NHS Trust, London, UK; 5 Department of Paediatric Endocrinology, University Hospital Southampton NHS Foundation Trust, Southampton, UK; 6 Department of Child Health, King’s College Hospital NHS Foundation Trust, London, UK; 7 Newcastle University and Great North Children’s Hospital, Newcastle upon Tyne, UK; 8 Bristol Royal Hospital for Children, University Hospitals Bristol, NHS Foundation Trust, Bristol, UK; 9 Faculty of Medicine, University of Southampton, Southampton, SO17 1BJ, UK; 10 Division of Population Medicine, School of Medicine, Cardiff University, Cardiff, UK; 11 Departments of Paediatrics, University College London Hospitals, London, UK; 12 Paediatric Endocrinology, Evelina London Children’s Hospital, Guy’s and St Thomas’ NHS Trust, London, UK; 13 Department of Oncology and Metabolism, University of Sheffield, Sheffield Children’s Hospital, Sheffield, UK; 14 Department of Paediatric Endocrinology, Royal Hospital for Children, NHS Greater Glasgow and Clyde, Glasgow, UK; 15 Northern Genetics Service, International Centre for Life, Newcastle, UK; 16 Developmental Biology & Medicine, Faculty of Biology, Medicine & Health, University of Manchester, and the Royal Manchester Children’s Hospital, Manchester University Hospital NHS Foundation Trust, Manchester, UK; 17 Wessex Regional Genetics Laboratory, Salisbury District Hospital, Salisbury, UK

**Keywords:** adrenal, adrenal insufficiency, Addison disease, genetics, NGS

## Abstract

**Context:**

Although primary adrenal insufficiency (PAI) in children and young people is often due to congenital adrenal hyperplasia (CAH) or autoimmunity, other genetic causes occur. The relative prevalence of these conditions is poorly understood.

**Objective:**

We investigated genetic causes of PAI in children and young people over a 25 year period.

**Design, Setting and Participants:**

Unpublished and published data were reviewed for 155 young people in the United Kingdom who underwent genetic analysis for PAI of unknown etiology in three major research centers between 1993 and 2018. We pre-excluded those with CAH, autoimmune, or metabolic causes. We obtained additional data from NR0B1 (DAX-1) clinical testing centers.

**Intervention and Outcome Measurements:**

Genetic analysis involved a candidate gene approach (1993 onward) or next generation sequencing (NGS; targeted panels, exomes) (2013-2018).

**Results:**

A genetic diagnosis was reached in 103/155 (66.5%) individuals. In 5 children the adrenal insufficiency resolved and no genetic cause was found. Pathogenic variants occurred in 11 genes: *MC2R* (adrenocorticotropin receptor; 30/155, 19.4%), *NR0B1* (DAX-1; 7.7%), *CYP11A1* (7.7%), *AAAS* (7.1%), *NNT* (6.5%), *MRAP* (4.5%), *TXNRD2* (4.5%), *STAR* (3.9%), *SAMD9* (3.2%), *CDKN1C* (1.3%), and *NR5A1*/steroidogenic factor-1 (SF-1; 0.6%). Additionally, 51 boys had *NR0B1* variants identified through clinical testing. Although age at presentation, treatment, ancestral background, and birthweight can provide diagnostic clues, genetic testing was often needed to define the cause.

**Conclusions:**

PAI in children and young people often has a genetic basis. Establishing the specific etiology can influence management of this lifelong condition. NGS approaches improve the diagnostic yield when many potential candidate genes are involved.

Primary adrenal insufficiency (PAI) is a potentially life-threatening condition that can be difficult to diagnose and that requires urgent treatment with glucocorticoid and sometimes mineralocorticoid replacement therapy [1]. PAI can occur across the lifespan and has many well established causes, such as congenital adrenal hyperplasia (CAH), autoimmune disorders (Addison disease) or physical damage of the adrenals (eg, hemorrhage, tumor) [1-4]. However, in many situations, a specific diagnosis is not readily reached.

The past 25 years have seen significant progress in our understanding of the genetic causes of PAI, especially for conditions presenting in children or in young people [4-8]. More than 30 different single gene disorders have now been reported with a diverse range of genetic inheritance patterns (recessive, dominant/de novo, X-linked, or even imprinted).

Establishing a specific diagnosis is important for understanding the natural history of a condition and how it might affect an individual, personalizing therapy (eg, the need for lifelong mineralocorticoid treatment), monitoring associated features (eg, puberty and gonadal function, renal), counseling about inheritance patterns and fertility, and identifying presymptomatic family members at risk. Many forms of PAI do not have diagnostic phenotypic or biochemical features, so genetic analysis is the only way to establish a precise diagnosis.

Genetic analysis traditionally required each candidate gene to be sequenced individually. This approach was time-consuming and expensive. More recently, next-generation sequencing (NGS) technologies have allowed parallel sequencing of many genes at the same time, either as a targeted panel of adrenal-related genes or as whole exome or genome sequencing. Recently, we have used a target-based NGS approach in a cohort study in Turkey of 95 children with PAI of unknown etiology (ie, CAH, autoimmune, metabolic causes excluded). With this approach, a genetic diagnosis was reached in 78/95 (82%) young people, involving just 9 genes [eg, *MC2R*, *NR0B1* (DAX-1), *STAR*, *CYP11A1*, *MRAP*, *NNT*, *ABCD1*, *NR5A1*, *AAAS*] [9]. However, the population studied had a high degree of consanguinity and marked genetic founder effects, so it is unclear if the findings can be translated to other populations in the world.

Here, we report our findings on the genetic analysis of children and young people with PAI of unknown etiology referred from within the United Kingdom (UK) to 3 major research centers and clinical testing centers for *NR0B1* (DAX-1) and provide a combined analysis of our published and unpublished experiences of diagnosing these conditions over 25 years.

## Materials and Methods

### Patient Cohort

The total cohort studied consisted of 155 children and young people (102 males, 48 females, five 46,XY girls) with PAI of unknown etiology living in the UK who were referred to our research groups (UCL GOS Institute of Child Health, London, UK; Queen Mary University, London, UK and Technische Universität Dresden, Dresden, Germany) for genetic analysis from several pediatric endocrine centers across the UK and where a genetic diagnosis was made between 1993 and 2018. Within the cohort there were 16 sibling pairs or familial pairs, 3 trios, and an extended family with 7 affected members.

All individuals included in the analysis had a provisional diagnosis of adrenal hypoplasia, familial glucocorticoid deficiency (FGD)/adrenocorticotropin (ACTH)-resistance, triple A syndrome/Allgrove syndrome (adrenal insufficiency, achalasia, alacrima), or just PAI with an unknown cause.

Subjects had undergone standard clinical and biochemical analysis to try to reach a diagnosis and were excluded if a specific cause was established [eg, CAH (21-hydroxylase deficiency, 11-hydroxylase deficiency, 3β-hydroxysteroid dehydrogenase deficiency type 2, 17α-hydroxylase deficiency, P450 oxidoreductase deficiency, aldosterone synthase deficiency), autoimmune adrenal disorders, metabolic dysfunction (eg, adrenoleukodystrophy), or physical causes]. Children with syndromic features, fetal growth restriction (intrauterine growth restriction), or genital anomalies (eg, hypospadias) were included.

Informed consent was obtained from affected individuals and/or their parents. Ethical approval for more recent studies was obtained from the London-Bloomsbury National Research Ethics Service committee (reference number 07/Q0508/24) and Outer North East London Research Ethics committee (reference number 09/H0701/12). Historical approvals for published data are detailed in the relevant publications (see Table 1).

**Table 1.** Overview of conditions, associated features, common presenting features and genetic variants identified

Gene (inheritance)	Potential associated features	N	Pathogenic variants (n)	Refs.^*a*^
*MC2R* (AR)	Possible tall stature, hyperpigmentation	30	p.S74I (20); *p.S74I/p.R128C*; *p.S74I/****p.L224R***; p.N81Kfs*3; p.D107N (3); p.R146H; p.R146H/p.V187Afs*29 (2); p.I154fs*248	[14-18]
*NR0B1* (X-linked)	Hypogonadotropic hypogonadism, infertility, possible early puberty	12	*NR0B1 deletion (3); contiguous gene deletion* ^*b*^ (2); p.W171*; p.L262Q; T265R; p.Y399*; p.H419Dfs*7 (2);***p.F448S***	[20-22,59]
*CYP11A1* (AR)	Possible gonadal steroid insufficiency	12	p.R120Q/p.Q395K; p.R120*/c.940G > A (2); c.426-2A > G/c.940G > A; c.790-802del/c.940G > A (3); p.A277Dfs*11/c.940G > A; p.I279Yfs*10/c.940G > A; p.S391 = /c.940G > A; p.R424*/c.940G > A; p.R451W	[9,11,25]
*AAAS* (AR)	Triple A syndrome (alacrima, achalasia, neurological features, dermatological features)	11	p.S382Rfs*33/p.W474*; p.H71Ifs*23/p.R230* (2); p.R286*/ ***c.525_545 + 4dupCCGTGTGTATAATGCCAGCAGGTGT*** ; p.R286*/p.R478*; p.Q145*/p.S263P; p.R230*/p.Q456*; p.G14Vfs*45 (2); **c.689 + 1G > C** (2)	[60-62]
*NNT* (AR)	Possible early puberty	10	p.R71*; p.Y201Kfs*1 (2); **c.599 + 2T > C**; p**.G255E** (2); p.G664R/p.T689Lfs*32 (2); p.G678R; p.L977P	[28,29]
*MRAP* (AR)	Hyperpigmentation	7	p.M1? (2); ***p.E28K****/c.106 + 2dupT*; c.106 + 2dupT (2); c.106 + 1G > C (2)	[31]
*TXNRD2* (AD)	Cardiac defects	7	p.Y447* (7)	[33]
*STAR* (AR)	Possible gonadal steroid insufficiency	6	**p.A10T**; p.V187M; ***p.G201D****/p.G221S* (2); p.G221D; p.T223Lfs*98	[35,37,38]
*SAMD9* ^*c*^ (AD/*de novo*)	MIRAGE syndrome (myelodysplasia, infection, restriction of growth, adrenal hypoplasia, genital phenotypes, enteropathy)	5	*p.R459Q*; *p.R982C*; *p.R982H*; *p.R1293Q*; *p.K1569N*	[39]
*CDKN1C* (AD, imprinted)	IMAGe syndrome (intrauterine growth restriction, metaphyseal dysplasia, adrenal hypoplasia, genitourinary anomalies)	2	*p.D274N*; *p.K278E*	[40]
*NR5A1* (AD/*de novo*)	Gonadal dysgenesis/steroid insufficiency	1	*p.G35E*	[41]

Italics indicates heterozygous (monoallelic) changes either as a dominant or compound heterozygous condition; bold indicates novel changes not reported previously by our groups or others; numbers in parentheses show the total number of individuals with this specific variant.

Abbreviations: AD, autosomal dominant; AR, autosomal recessive.

^*a*^References indicate our previous reports including these findings or other groups’ reports of these genetic variants (see supplementary table in [13]).

^*b*^One girl with a Xp21 contiguous gene deletion syndrome expressed a phenotype due to skewed X-inactivation.

^*c*^For SAMD9 the primary genetic change is shown, although several children had developed somatic revertant events such as monosomy 7 or second loss-of-function variants in SAMD9.

An additional cohort of children were identified (n = 51) who had been diagnosed with X-linked adrenal hypoplasia due to pathogenic variants in *NR0B1* (DAX-1) through clinical genetic testing pathways. This supplementary group was important to consider as clinical testing became more widespread in the 2000s, and we did not want the NR0B1 group to be underrepresented in the cohort, as it is an important diagnosis to make. Data were obtained primarily from Wessex Regional Genetics Laboratory and from the Northern Genetics Service, Newcastle. Individuals with *NR0B1* mutations in the research cohort and the clinical testing group were independent.

### Genetic Analysis: Sanger Sequencing

In the first 20 years of the study (1993-2012), genetic analysis was mostly undertaken by Sanger sequencing using a candidate gene approach. The main genes analyzed using this method at the time were *MC2R*, *MRAP*, *AAAS*, *NNT*, *NR0B1* (DAX-1), *NR5A1* (SF-1), *CDKN1C*, *STAR*, and *CYP11A1*, and key findings and sequencing methods were published where appropriate. Subsequently, a more refined Sanger sequencing approach was used for initial analysis (*MC2R*, *MRAP* exon 3, *CYP11A1* exon 5, *STAR* exons 4-5, *NR0B1*).

### Genetic Analysis: HaloPlex-Targeted Capture and NGS

Since 2013, a NGS strategy was used to try to reach a specific genetic diagnosis in 75 patients in whom no diagnosis had been reached, either using a HaloPlex-targeted panel or exome sequencing.

#### HaloPlex targeted capture

A HaloPlex DNA target enrichment panel was designed using SureDesign (Agilent Technologies Inc., Santa Clara, CA, USA) to include all known genes involved in PAI at the time (2013) [9,10].

Patient DNA was extracted from leukocytes using standard methods. Batches of 24 samples were run together, including 23 patient samples and one enhanced control DNA for quality control. Genomic DNA samples (225 ng) were processed for Illumina Sequencing according to the “HaloPlex Target Enrichment System” protocol (Version D.5, Agilent Technologies Inc.) and as described previously [9]. Resultant libraries were then subjected to NGS using an Illumina MiSeq platform (Illumina Inc., San Diego, CA, USA). Raw FASTQ files were analyzed using SureCall (v3.0.1.4) software (Agilent Technologies, Inc.) and Ingenuity Pathway Analysis (Qiagen Bioinformatics, Inc., Redwood City, CA, USA). Aligned BAM files were also visualized manually to review for potential whole gene or single exon deletions.

#### Whole exome sequencing

Exome sequencing was undertaken using the Agilent SureSelect all exon V4 capture and paired-end (2 × 100) sequencing on an Illumina HiSeq 2000 at Otogenetics (Norcross, GA, USA). Alignment, variant calling, and annotation were performed by DNAnexus (DNAnexus Inc., Mountain View, CA, USA) using the DNA nexus Classic platform or Ingenuity Variant Analysis. Raw data were also reanalyzed by BWA-MEM FastQ Readmapper, variants called using Vendor Human Exome GATK-Lite (Unified Genotyper) and analyzed in Ingenuity Variant Analysis using filtering criteria reported previously [11].

### Variant Validation

Sanger sequencing was used to confirm the presence of key variants identified by NGS. Regions of interest were polymerase chain reaction amplified and sequenced using a Big Dye Terminator v.1.1 Cycle Sequencing Kit (Applied Biosystems) and ABI3130 sequencer (Applied Biosystems), and visualized using Sequencher v5.2.4 (Gene Codes Corporation). Population genomic variation control data were obtained from Genome Aggregation Database (gnomAD, Cambridge, MA, USA; https://gnomad.broadinstitute.org) [12].

### Functional Prediction of Genetic Variants

The predicted functional effects of specific variants were performed using Ensembl Variant Effect Predictor, SIFT, PolyPhen2, and MutationTaster algorithms (https://www.ensembl.org/info/docs/tools/vep/index.html; http://sift.jcvi.org/; http://genetics.bwh.harvard.edu/pph2/; http://www.mutationtaster.org/, respectively).

### Statistical Analysis

Chi-square tests for sex differences were performed using GraphPad Prism version 8.4.3 for Windows (GraphPad Software, San Diego, CA, USA; www.graphpad.com).

## Results

### Cohort Results

**Figure 1.**
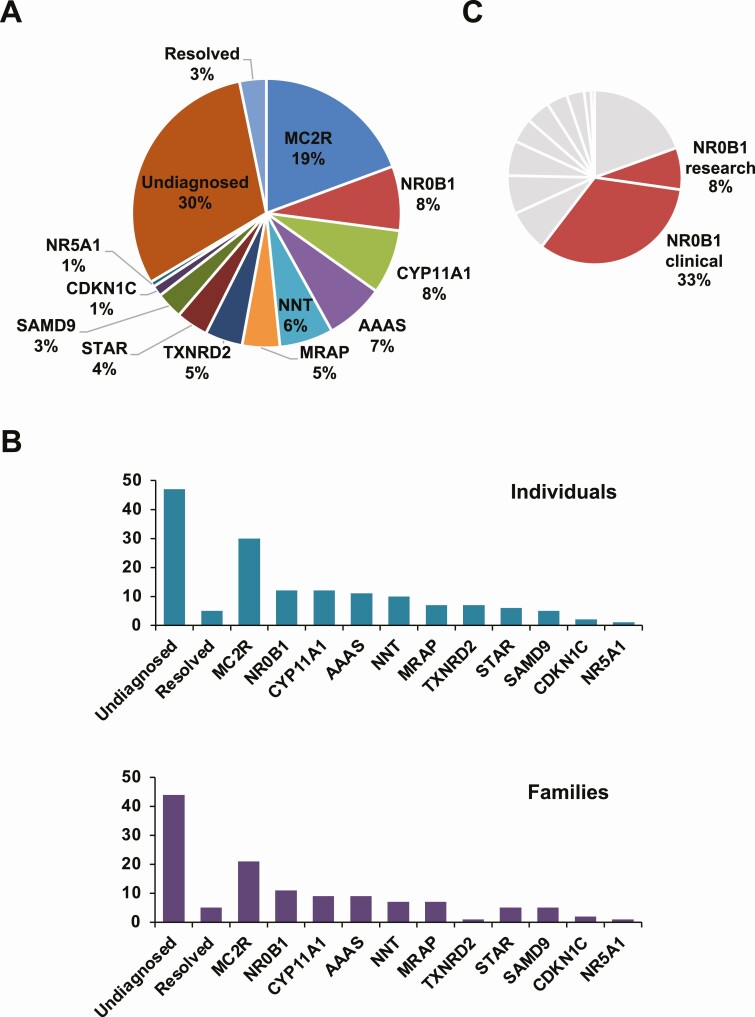
Overview of the study cohort and genetic diagnoses reached. (A) Relative percentages of each diagnosis for the research cohort in relation to undiagnosed and resolved categories (n = 155). (B) Numbers of individuals (n = 155) with each genetic diagnosis (upper panel) and number of different affected families (n = 127) (lower panel). Note: data for X-linked adrenal hypoplasia (DAX-1/*NR0B1*) identified through clinical services not included here. (C) Relative percentages of *NR0B1* diagnoses (research and clinical) (combined n = 63) in relation to all diagnosed individuals (n = 154). Data represent the number of individuals, not families.

Within the entire cohort, a specific genetic diagnosis was reached in 103/155 (66.5%) individuals (Fig. 1A). No specific genetic diagnosis was found in 47/155 (30.3%) individuals (29 had NGS; 18 had candidate gene sequencing). The adrenal insufficiency resolved and steroid replacement treatment was gradually withdrawn during the course of this study in 5/155 (3.2%) children, including in 1 boy 13 years after initial treatment. Of note, no genetic cause was found in this group.

**Figure 2.**
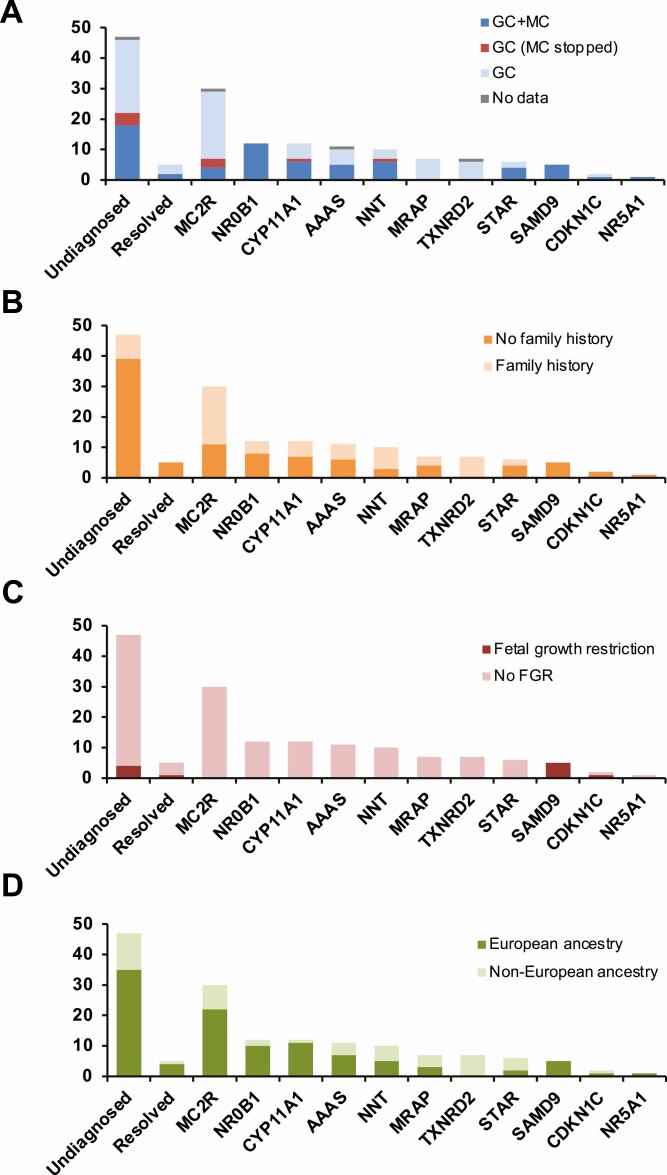
Key features of each diagnostic group. (A) Steroid replacement received. (B) Presence or absence of a positive family history of adrenal insufficiency. (C) Presence or absence of fetal growth restriction (FGR) (<2.5 kg at term). (D) Ancestry. European was defined as of a White European background compared to other backgrounds such as Asian, Asian British, African, or Black British. Data for X-linked adrenal hypoplasia (DAX-1/*NR0B1*) identified through clinical services not included here or in subsequent figures. Abbreviations: GC, glucocorticoids; MC, mineralocorticoids.

**Figure 3.**
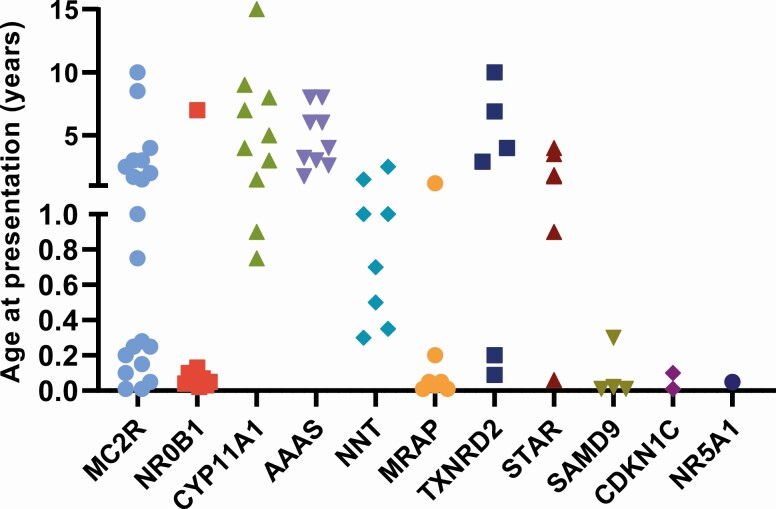
Age of main presentation of PAI in the different groups with a genetic diagnosis. Note the nonlinear age scale.

Pathogenic variants were found in 11 different genes (Fig. 1A and B; Tables 1 and 2; also see supplementary table in [13]). Related characteristics for each genetic diagnosis (eg, treatment, family history, fetal growth restriction, ancestry) are shown in Fig. 2A-2D, and age at presentation of the adrenal insufficiency in each subgroup is shown in Figure 3.

**Table 2.** Novel pathogenic variants identified and their predicted pathogenicity

Gene	Sequence variation		gnomAD Allele Frequency	VEP		SIFT	PolyPhen-2	Mutation Taster
	cDNA	Protein		SIFT	PolyPhen			
*MC2R*	c.243delT	p.N81Kfs*3	nd	Frameshift		na	na	Disease causing
	c.671T > G	p.L224R	4/282270	0	0.945	0	0.995	Disease causing
*NR0B1*	c.1343T > C	p.F448S	nd	0	0.8	0.001	0.981	Disease causing
AAAS	c.525_545 + 4dupCCGTGTGTA TAATGCCAGCAGGTGT	na	3/281760	Splice defect		na	na	Disease causing
	c.689 + 1G > C	na	nd	Splice donor		na	na	Disease causing
*NNT*	c.599 + 2T > C	na	nd	Splice donor		na	na	Disease causing
	c.764G > A	p.G255E	nd	0	0.988	0.001	0.999	Disease causing
*MRAP*	c.82G > A	p.E28K	3/251322	0.04	0.998	0.071	1	Disease causing
*STAR*	c.28G > A	p.A10T	nd	0.01	0.503	0.014	0.95	Disease causing
	c.602G > A	p.G201D	3/282724	0	0.944	0	0.992	Disease causing
	c.666delC	p.T223Lfs*98	nd	Frameshift		na	na	Disease causing

Abbreviations: na, not applicable; nd, not detected; VEP, Ensembl Variant Effect Predictor.

An additional 51 boys were identified with pathogenic variants in *NR0B1* (DAX-1) through clinical testing pathways (Fig. 1C).

### MC2R

One of the most prevalent conditions diagnosed was FGD type 1 due to disruption of the ACTH receptor melanocortin receptor-2 (*MC2R*) [14-18]. Pathogenic variants were identified in 19.4% (30/155) of the entire cohort. This condition was most often (20/30) due to the p.S74I variant found in individuals of European ancestry from Ireland and Scotland [19]. Two siblings had a p.V187Afs*29 (c.560delT) variant, which is now being seen in Turkey and neighboring regions [9,17]. Most young people were treated with glucocorticoids alone. Seven children were treated with additional mineralocorticoids, but 3 of these were able to stop the mineralocorticoid treatment once the diagnosis had been made (Fig. 2A).

### NR0B1 (DAX-1)

Disruption of *NR0B1* (DAX-1), causing X-linked adrenal hypoplasia congenita (AHC) was found in 7.7% (12/155) of children in the research cohort [20,21]. Presentation was mostly in the newborn period with salt-losing adrenal insufficiency in boys, but sometimes more insidiously in later childhood (Fig. 3). Analysis showed that 7 boys had missense, frameshift, or stop gain mutations, whereas 3 had localized deletions of the *NR0B1* gene itself. An Xp21 contiguous gene deletion syndrome including glycerol kinase deficiency and Duchenne muscular dystrophy was also diagnosed in 2 children; surprisingly, 1 of these was a girl who had a large Xp21 deletion and expressed the phenotype due to skewed X inactivation [22]. All boys of a pubertal age have shown evidence of hypogonadotropic hypogonadism and required pubertal induction.

An additional 51 boys from 42 families were identified with pathogenic *NR0B1* variants through clinical testing pathways. Taken together with the research cohort (*NR0B1*, n = 12), 63 children had a diagnosis of X-linked AHC out of a total of 154 children with a positive genetic diagnosis (103 research cohort, 51 *NR0B1* clinical cohort). These combined data suggest that X-linked AHC represented the largest diagnostic group overall (63/154, 40.9%) (Fig. 1C).

### CYP11A1

*CYP11A1* mutations were found in 7.7% (12/155) of the cohort. *CYP11A1* encodes the enzyme P450 side-chain cleavage enzyme (P450scc), which converts cholesterol to pregnenolone. P450scc is needed for the synthesis of all adrenal and gonadal steroids and severe disruption of this protein causes early-onset salt-losing adrenal insufficiency and impaired virilization/46,XY DSD [23,24]. Most of the patients in our cohort had partial loss of *CYP11A1* activity and often presented in childhood with signs of glucocorticoid insufficiency, such as ketotic hypoglycemia (Fig. 3). Approximately half of them had evidence of mildly impaired zona glomerulosa function and were treated with mineralocorticoids (Fig. 2A). Ten of the 12 individuals were from a UK background and had the c.940G > A variant in compound heterozygosity with another severely disruptive change [11,25,26]. One child was compound heterozygous for p.R120Q and p.Q395K and 1 individual, whose family were from central Turkey, was homozygous for p.R451W, a variant that is prevalent in that region [9].

### AAAS

Triple A syndrome (Allgrove syndrome) is a rare autosomal recessive multisystem disease characterized by adrenal insufficiency, alacrima, and achalasia caused by pathogenic variants in *AAAS* [27]. In this cohort, 7.1% (11/155) of children had *AAAS* variants. Most of these were nonsense or frameshift changes, with 1 novel, homozygous splice site disruption (c.689 + 1G > C) found in 2 cousins and a second novel splice site mutation (c.525_545 + 4dupCCGTGTGTATAATGCCAGCAGGTGT) with predicted loss of protein function. Children with triple A usually developed adrenal insufficiency in mid-childhood (age range 1.75-8 years) (Fig. 3) and often had other features such as alacrima, achalasia, and neurological symptoms at this time. Approximately half required treatment with both glucocorticoids and mineralocorticoids, whereas half received glucocorticoids alone.

### NNT

Defects in nicotinamide nucleotide transhydrogenase (*NNT*) were found in 10/155 (6.5%) children who presented between 4 months and 2.5 years of age [28,29]. This protein is involved in regulating mitochondrial oxidative stress. Three children experienced early puberty, which has been reported recently as a potential associated feature of this condition [30]. One child of Pakistani ancestry was homozygous for a stop gain (p.R71*); this variant has been previously identified in a child living in Australia whose mother originated from this region [29].

### MRAP

Disruption of the melanocortin 2 receptor-associated protein (*MRAP*) causes FGD2 and was found in 7/155 (4.5%) of individuals [31]. Most of these changes affected translation initiation (p.M1?) or splicing events, as reported previously, and usually occurred in children who presented in the neonatal period or first 3 months of life [32].

### TXNRD2

A similar number of individuals (4.5%) were found to have adrenal insufficiency due to a homozygous stop gain mutation (p.Y447*) in thioredoxin reductase 2 (*TXNRD2*) (Fig. 1B) [33]. However, individuals were all from a single Kashmiri kindred (Fig. 1C). No pathogenic variants in TXNRD2 were found in other patients.

### STAR

Similar to *CYP11A1*, defects in steroidogenic acute regulatory protein (*STAR*) can affect both adrenal and gonadal steroid synthesis. Severe disruption of STAR causes classic congenital lipoid adrenal hyperplasia (CLAH) [34]. This condition was diagnosed in a 46,XY girl with early-onset salt-losing adrenal insufficiency due to a homozygous frameshift mutation. Five others who presented between 9 months of age and 4 years with predominant glucocorticoid insufficiency had nonclassic CLAH, although mineralocorticoids were given to most of them [35-38]. Nonclassic CLAH was not associated with obvious genital anomalies.

### SAMD9 and CDKN1C

Five children (5/155, 3.2%) had de novo, gain of function mutations in sterile alpha motif domain-containing 9 (*SAMD9*) [39]. They were all born preterm and had fetal growth restriction, together with other features of MIRAGE syndrome (myelodysplasia, infection, restriction of growth, adrenal hypoplasia, genital phenotypes, enteropathy). Two other children in the cohort had gain of function changes in the proliferating cell nuclear antigen–binding domain of cyclin dependent kinase inhibitor 1C (*CDKN1C*) associated with IMAGe syndrome (intrauterine growth restriction, metaphyseal dysplasia, adrenal hypoplasia, genitourinary anomalies) [40]. *CDKN1C* is an imprinted gene. In 1 child, there was no marked growth restriction and features were mild.

### NR5A1 (SF-1)

Only 1 individual was found to have a pathogenic variant in *NR5A1*, encoding steroidogenic factor-1 (SF-1) [41]. This 46,XY child presented with salt-loss and a female phenotype. No variants in *NR5A1* were found to cause PAI alone.

### Sex Differences

**Figure 4.**
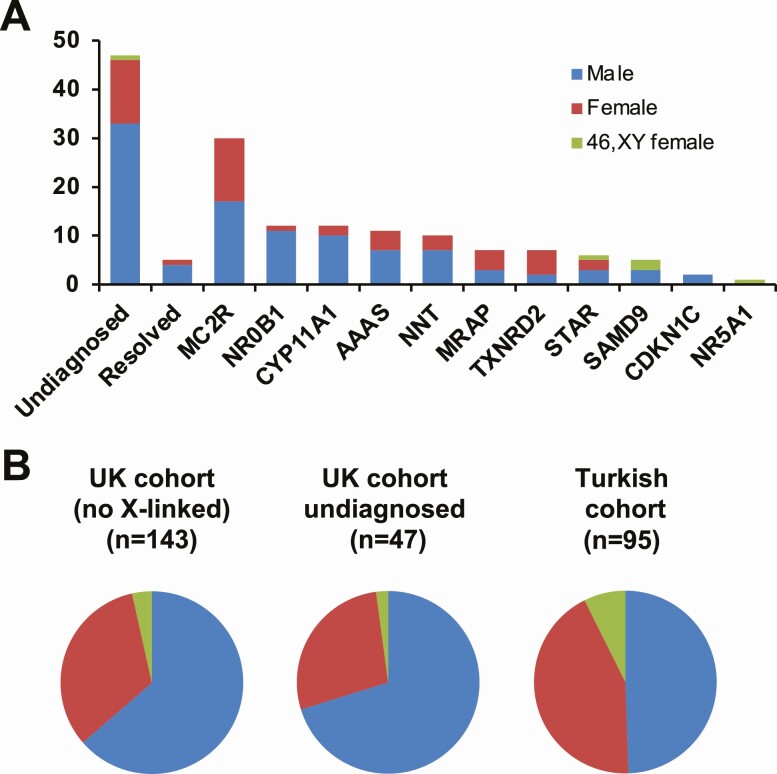
Sex differences in the cohort. (A) Number of males, females and 46,XY females in each group. (B) Relative proportions of males, females and 46,XY females in the total UK cohort with X-linked conditions removed (left chart), the “undiagnosed” UK cohort (center chart) and previously published Turkish cohort (right chart).

Analysis of sex differences within the cohort revealed that there was an excess of boys in the UK cohort (102/155, 65.8%; χ ^2^ = 22.17, *P* < 0.001) (Fig. 4A). This observation persisted even when individuals with X-linked adrenal hypoplasia (NR0B1) were removed from analysis (91/143, 63.6%; χ ^2^ = 16.20, *P* < 0.001) (Fig. 4B). Indeed, the sex-differences were even more marked in the “undiagnosed” group (33/47, 70.2%; χ ^2^ = 8.7, *P* = 0.0032) and in the group where PAI had resolved (4/5) (Fig. 4B). This observation contrasts with data from the Turkish cohort where sex-differences were not seen (47/95, 49.5%; χ ^2^ = 0.4, *P* = 0.5) [9].

## Discussion

PAI is an extremely important diagnosis to make and establishing a specific genetic diagnosis can have important implications. The most common cause of PAI in infants and children is CAH, accounting for approximately 80% to 85% of PAI in studies to date [2,42-44]. CAH occurs most often due to 21-hydroxylase deficiency, and the diagnosis can usually be confirmed by biochemical and genetic analysis. Several “hotspots” of other forms of CAH exist (eg, 11β-hydroxylase deficiency in Jewish populations from Morocco); indeed, congenital lipoid adrenal hyperplasia due to disruption of STAR is particularly prevalent in South Korea and Japan [2,6]. Other well-recognized causes of PAI include autoimmune disorders (eg, autoimmune polyglandular syndrome type 1/*AIRE*; autoimmune Addison disease in teenagers) and established but rare metabolic disorders (eg, Wolman disease, Zellweger spectrum, mitochondrial disorders, adrenoleukodystrophy) [4]. Of these metabolic conditions, X-linked adrenoleukodystrophy is an important diagnosis to make in boys, and very-long change fatty acid analysis should be considered. However, in many children a specific cause of PAI is not obvious and additional genetic investigation is needed.

Here, we have combined published and unpublished data over a 25-year period from 3 major research centers investigating childhood PAI in the UK to provide insight into the genetic basis of some of the rarer forms of PAI in children and young people, where CAH, autoimmune and metabolic causes had been excluded. A diagnosis was reached in 66.5% of the cohort. This figure is lower than in the recent Turkish study (82%) where there was higher consanguinity but still represents a significant diagnostic yield for monogenic disorders, especially as CAH and other metabolic conditions had been excluded [9]. Furthermore, 51 boys from 42 independently ascertained families had *NR0B1* variants identified through clinical testing pathways, so the UK diagnostic yield is likely to be even higher.

The proposed benefits of achieving a specific genetic diagnosis include being able to counsel the family appropriately and identifying at risk relatives before the onset of an adrenal crisis; understanding the natural history of different conditions and monitoring for the development of known associated features; and, in some situations, being able to modify treatment strategies. Within this cohort there are several examples of each of these situations.

First, in several children with familial forms of X-linked AHC (*NR0B1*/DAX-1), MC2R defects or TXNRD2 disruption, we were able to make a diagnosis of adrenal insufficiency early on in the presentation or whilst the child was presymptomatic. In 1 family (*NR0B1*), steroid treatment was commenced as sodium started to fall in the first week of life, but before the onset of a severe salt-losing state or extreme hyperkalemia. In another family *(TXNRD2)*, symptoms such as malaise were linked to PAI rather than being overlooked. PAI can be a difficult diagnosis to make in any child presenting for the first time as features can be nonspecific. We have previously shown in families where there are 2 brothers with X-linked AHC due to *NR0B1*/DAX-1 mutations that the second boy is typically diagnosed at a younger age than the first, often because of heightened awareness [45]. By combining genetic testing with biochemistry, a presymptomatic diagnosis should be possible. Indeed, in some situations a child’s biochemistry will be within normal limits early on and genetic analysis may be the only diagnostic test.

**Figure 5.**
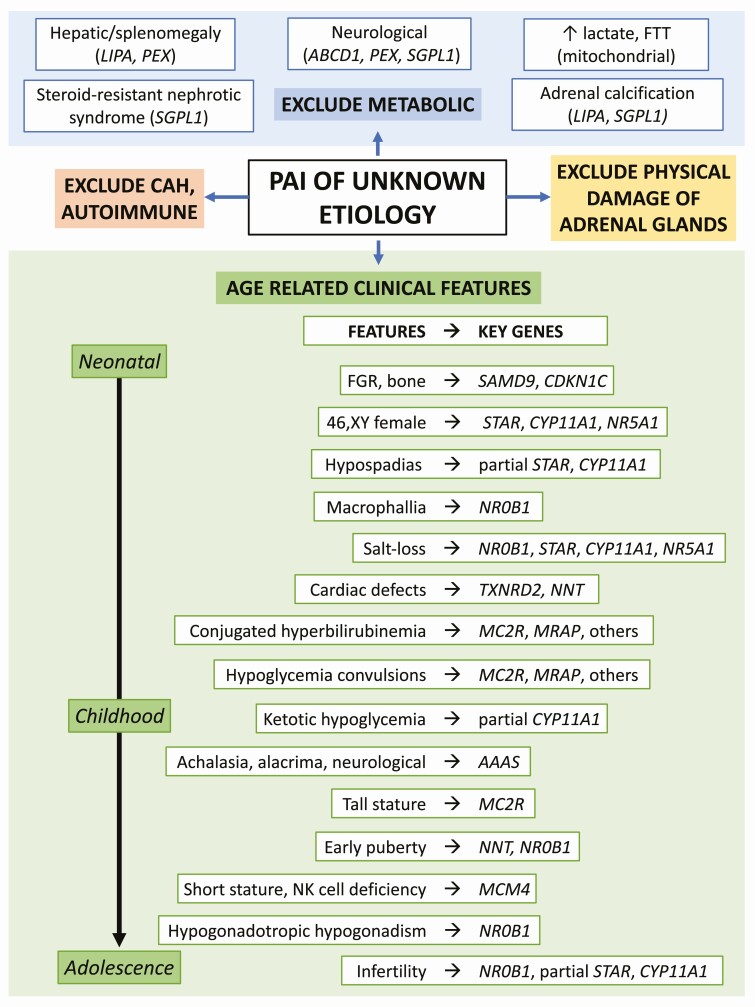
Selected clinical features that might be associated with different genetic etiologies of PAI. Abbreviations: CAH, congenital adrenal hyperplasia; FGR, fetal growth restriction; FTT, failure to thrive.

Second, detecting potential associated features is important (Fig. 5). For example, boys with X-linked adrenal hypoplasia (*NR0B1*/DAX-1) are at risk of developing hypogonadotropic hypogonadism in adolescence, whereas there is a potential risk of long-term sex steroid insufficiency or hypofertility with partial defects in STAR or *CYP11A1*/P450scc [7,25,35,36,46]. Appropriate monitoring and counseling can be provided, and cryopreservation of sperm might be considered. There are reports of testicular adrenal rest tumors with poor control in some of these conditions too [46,47]. Patients with triple A syndrome often present with additional features such as alacrima (93% of all patients, 64% of them within the first 36 months of life), achalasia (86% of all patients with an average age of 6.4 years), dermatological symptoms (71% of all patients), and neurological symptom (72% of all patients) [48]. Peripheral muscle weakness, nasal speech, and hyperreflexia without other evidence of spasticity are pathognomonic signs of triple A syndrome. In addition, growth-restricted infants diagnosed with MIRAGE syndrome or IMAGe syndrome should have appropriate monitoring of associated features. In some situations children with MIRAGE syndrome may require stem cell transplant if monosomy 7-related myelodysplastic syndrome and subsequent hematopoietic changes develop [39,49]. Finally, in conditions with no strong associated features (eg, MC2R, MRAP) reassurance can be given.

Several examples also emerged where treatment was modified or personalized based on genetic analysis. Several children with ACTH resistance (*MC2R* mutations) were started on mineralocorticoid treatment at diagnosis, but this was withdrawn later in life when the diagnosis was made and mineralocorticoid requirements are less [17]. Adequate salt and fluid intake are still recommended during heat and exertion though. Also, there can be a tendency to try to suppress ACTH concentrations in some forms of ACTH resistance, which can lead to overtreatment with replacement glucocorticoids, and an awareness of the diagnosis can help in this regard. Of particular interest is the small group of children who were able to stop steroid hormone replacement and recover adrenal function during the course of this work. None of them was found subsequently to have a genetic diagnosis in these key genes. Diagnosing adrenal insufficiency can be difficult especially in a sick child with hyponatremia or hypoglycemia, and if a full diagnostic work-up including ACTH measurements is not available, children may have been started on treatment at the time, erring on the side of caution. Readdressing the diagnosis can be difficult once established on steroid treatment, as the hypothalamo-pituitary-adrenal axis may be suppressed for years, even if relatively low-maintenance doses have been used [50]. While we would not support generalized withdrawal of treatment when a genetic cause is not found, especially as this is research rather than diagnostic testing, it could be envisaged that a lower threshold for a trial off treatment would be considered in some situations, especially where evidence for initial diagnosis is not robust, and is based on incomplete biochemical data. Being able to make any genuine change in treatment for children and young people has major implications, as this is a lifelong condition.

With the emergence of NGS approaches, either as panels of genes or whole exomes, it is now often cheaper and quicker to adopt this approach rather than a candidate gene approach. In the UK, clinical testing with panels has now been introduced. However, in some settings, resources are limited and more focused genetic analysis may be needed. By assimilating data over a 25-year period, some useful insights have emerged. For example, X-linked adrenal hypoplasia usually affects boys and often presents with salt-loss in the first 2 months of life, whereas glucocorticoid insufficiency often presents with prolonged neonatal jaundice or hypoglycemic convulsions. Defects in MRAP tend to present in early life, whereas presentation with NNT mutations is usually between 6 months and 4 years of age, and triple A syndrome more often in mid-childhood (Fig. 3). Partial loss of CYP11A1 or STAR can present in childhood with ketotic hypoglycemia. These data are largely supportive of our findings in the Turkish cohort study [9].

Furthermore, ancestral background is emerging as an important predictor of genetic etiology in some children. For example, the p.S74I variant in MC2R is by far the most common change seen in our UK population with FGD, which likely arises from an Irish/Scottish founder effect [19]. The c.940G > A variant in *CYP11A1* causing misplicing and a partial phenotype was found in 10 patients of UK ancestry and has been reported in families from France, Spain, the United States, Canada, and Australia recently [25,26,46]. A p.R451W variant in CYP11A1 was found in a child born to a family originating from central Turkey; reports have also described this change in Germany in a family of central Turkish origin [9,51]. The c.560delT change in *MC2R* also has a founder effect from Turkey and neighboring regions [9,17]. Finally, the p.R71* stop gain in NNT in a family of Pakistani origin living in Scotland has also been recently reported by us in a woman living in Australia who also originated from this region [29]. Therefore, a careful family history is always important when assessing a child with PAI.

This study has several limitations. First, this cohort represents those children and families sent to us for genetic analysis and does not represent a cross-sectional cohort or means of calculating incidence or prevalence of these conditions. We were likely to be biased in receiving more familial cases rather than sporadic, and in recent years clinical genetic testing for conditions such as X-linked adrenal hypoplasia (*NR0B1*/DAX-1) has become more widely available, meaning that this group in particular may be underrepresented. We addressed this to some extent by identifying clinically diagnosed children with pathogenic variants in *NR0B1*, but it was not possible to combine these data with the research cohort as we lacked details about specific characteristics for this group, as well as for those children who tested negative for DAX-1. We did not find any pathogenic variants in MCM4 (adrenal insufficiency, short stature, natural killer cell deficiency), which, to date, has only been reported in the Irish Traveller population, nor in SGPL1 (adrenal insufficiency, steroid-resistant nephrotic syndrome, ichthyosis, other features) in this UK resident population, although not all children had full analysis with panels or exomes [52,53]. Very recently described causes of adrenal insufficiency (such as POLE1 deficiency) were not considered, although none of our cohort had clear phenotypic features of this syndrome [54]. Finally, adrenal insufficiency can also arise from nongenetic causes in some situations, such a bilateral adrenal hemorrhage [55]. Adrenal imaging is not always undertaken or features may resolve, so potentially this is underdiagnosed.

Within our undiagnosed cohort, it is possible there are new genetic causes of adrenal insufficiency to be discovered or we may have overlooked deep intronic changes that are emerging from our work as a more common cause of gene disruption than initially thought [29,56,57]. It is also interesting that we see a sex-bias in the UK-based population, with more boys being diagnosed with PAI than girls, especially in the undiagnosed group (Fig. 4). This suggests that an X-linked phenomenon might exist that we are overlooking, such as a new genetic cause or alteration in a regulatory region of a key gene such as *NR0B1* [58]. We hypothesize that there could also be sex differences in adrenal function in males and females, which could manifest as a susceptibility to transient adrenal hypofunction in the newborn period in boys and diagnosis with PAI. Although numbers are small, 4/5 children where PAI resolved were boys. This hypothesis requires further investigation, particularly linked to developmental sex differences [10]. We have recently shown how the phenotype associated with MIRAGE syndrome can be influenced by developmental events such as somatic reversion [39]. It is also possible that genetic plasticity and sex differences can influence adrenal function across the life course in other conditions.

In summary, this retrospective observational study shows that genetic analysis can help reach a specific diagnosis in almost 70% of children and young people with PAI of unknown etiology, with potential implications for long-term counseling and management. Taken together with CAH and other established causes of adrenal dysfunction, it is now likely that a specific diagnosis can be reached in the vast majority of children and young people with PAI. The adoption of PAI into clinical genetic testing platforms in countries such as the UK should help considerably in improving the speed and rate of diagnosis for these conditions in the future.

## Data Availability

Some or all data sets generated during and/or analyzed during the current study are not publicly available but are available from the corresponding author on reasonable request.

## Additional Information

***Disclosures:*** The authors have nothing to declare.
